# Blood pressure and expression of microRNAs in whole blood

**DOI:** 10.1371/journal.pone.0173550

**Published:** 2017-03-09

**Authors:** Zhou Zhang, Brian Thomas Joyce, Jacob K. Kresovich, Yinan Zheng, Jia Zhong, Ruchi Patel, Wei Zhang, Lei Liu, Chang Dou, John P. McCracken, Anaité Díaz, Valeria Motta, Marco Sanchez-Guerra, Shurui Bian, Pier Alberto Bertazzi, Joel Schwartz, Andrea A. Baccarelli, Sheng Wang, Lifang Hou

**Affiliations:** 1 Driskill Graduate Program in Life Sciences, Feinberg School of Medicine, Northwestern University, Chicago, IL, United States of America; 2 Department of Preventive Medicine, Northwestern University Feinberg School of Medicine, Chicago, IL, United States of America; 3 Division of Epidemiology/Biostatistics, School of Public Health, University of Illinois-Chicago, Chicago, IL, United States of America; 4 Institute for Public Health and Medicine, Feinberg School of Medicine, Northwestern University, Chicago, IL, United States of America; 5 Department of Environmental Health, Harvard School of Public Health, Boston, MA, United States of America; 6 Robert H. Lurie Comprehensive Cancer Center, Feinberg School of Medicine, Northwestern University, Chicago, IL, United States of America; 7 Department of Safety Engineering, China Institute of Industrial Relations, Beijing, China; 8 Center for Health Studies, Universidad del Valle de Guatemala, Guatemala City, Guatemala; 9 Department of Clinical Sciences and Community Health, University of Milan and IRCCS Fondazione Ca’ Granda Ospedale Maggiore Policlinico, Milan, Italy; 10 Department of Developmental Neurobiology, National Institute of Perinatology, CDMX, Mexico City, Mexico; 11 Department of Environmental Health Sciences, Columbia University Mailman School of Public Health, New York, NY, United States of America; 12 Department of Occupational and Environmental Health, Peking University Health Science Center, Beijing, China; University of Utah School of Medicine, UNITED STATES

## Abstract

**Background:**

Blood pressure (BP) is a complex, multifactorial clinical outcome driven by genetic susceptibility, behavioral choices, and environmental factors. Many molecular mechanisms have been proposed for the pathophysiology of high BP even as its prevalence continues to grow worldwide, increasing morbidity and marking it as a major public health concern. To address this, we evaluated miRNA profiling in blood leukocytes as potential biomarkers of BP and BP-related risk factors.

**Methods:**

The Beijing Truck Driver Air Pollution Study included 60 truck drivers and 60 office workers examined in 2008. On two days separated by 1–2 weeks, we examined three BP measures: systolic, diastolic, and mean arterial pressure measured at both pre- and post-work exams for blood NanoString nCounter miRNA profiles. We used covariate-adjusted linear mixed-effect models to examine associations between BP and increased miRNA expression in both pooled and risk factor-stratified analyses.

**Results:**

Overall 43 miRNAs were associated with pre-work BP (FDR<0.05). In stratified analyses different but overlapping groups of miRNAs were associated with pre-work BP in truck drivers, high-BMI participants, and usual alcohol drinkers (FDR<0.05). Only four miRNAs were associated with post-work BP (FDR<0.05), in ever smokers.

**Conclusion:**

Our results suggest that many miRNAs were significantly associated with BP in subgroups exposed to known hypertension risk factors. These findings shed light on the underlying molecular mechanisms of BP, and may assist with the development of a miRNA panel for early detection of hypertension.

## Introduction

Hypertension (HTN), defined as systolic blood pressure (SBP) above 140 mmHg or diastolic blood pressure (DBP) above 90 mmHg, is a major public health concern worldwide [1, 2]. Despite our understanding of the disease and availability of treatments, 80 million adults in the US [3] as well as 266 million adults in China [4] suffer from HTN, indicating a substantial unrelieved public health burden. With primary prevention efforts (e.g., the DASH diet) [5, 6] largely unsuccessful in populations in recent decades, secondary prevention through earlier disease detection may aid reducing HTN-related health disparities and economic burdens. In particular analyses of whole blood have found shifts in environment-induced gene expression that presage systemic pro-inflammatory processes [7–9] and can predict future cardiovascular disease risk [10]. Greater insight into molecular mechanisms related to elevated blood pressure (EBP), a physiological event related to clinical HTN, can assist in addressing its current unmet public health burden.

Since EBP results from a set of complex genetic, pathophysiological, and environmental factors [11] post-translational modifications are a natural candidate for biomarker studies of hypertension risk factors and early detection [11–13]. Post-translational modifications to gene expression include DNA methylation, histone modification, and microRNAs (miRNAs), and can all functionally alter gene expression without changing the underlying DNA sequence [13, 14]. miRNAs are small (20–24 base) nucleotides that induce messenger RNA (mRNA) cleavage or reduce translation to regulate gene expression [15–18], thus having a potentially profound impact on diseases including HTN. Studies have connected handfuls of miRNAs such as the miRNA130/301 family [19] to HTN via pathways such as promoting vasoconstriction and thus increasing pulmonary BP [20]. Due to these associations and the stability of miRNAs, researchers have previously suggested their potential use as biomarkers for HTN [19–22]. However, many of the specific biological mechanisms underlying the relationship between miRNAs and EBP or EBP-related risk factors have yet to be elucidated.

Our group previously reported that traffic-related exposure to ambient PM_10_ (particulate matter ≤10 μm) affects blood pressure (BP) [23], and that this exposure was associated with the expression of human miRNAs [24]. As an additional component to this research we investigated miRNA profiles in blood leukocytes and their relation to BP in 120 workers in urban Beijing [25]. Because evidence suggests that BP varies throughout the day [26, 27], our objective was to explore the separate relationships between BP measured pre- and post-work and miRNA expression levels.

## Materials and methods

### Study participants

The Beijing Truck Driver Air Pollution Study was conducted between June 15 and July 27, 2008 in two groups highly exposed to air pollution: 60 truck drivers and 60 office workers [23]. Both of these occupation groups were matched by sex, smoking status, education, and age (+5 years). Each participant was examined twice each on two days one to two weeks apart, for a total of two examination days and four examinations per participant to allow for short-term variations in BP. Blood samples were collected at the end of each examination day only, allowing for two miRNA measures per participant (240 total). We used a self-administered questionnaire to collect detailed information on demographics and lifestyle as well as time-varying factors (e.g., smoking) which were self-reported both for past patterns and day of examination data (i.e., smoking status and number of cigarettes smoked on the exam day respectively). Daily temperature and dew point data for Beijing were obtained from the National Oceanic and Atmospheric Administration [28]. Individual written informed consent was obtained from all participants prior to enrollment in the study. Institutional Review Board or equivalent approval at the participating institution (i.e., Harvard School of Public Health, Northwestern University, and Peking University Health Science Center) was obtained prior to study participant recruitment.

### miRNA measurement

A total of 240 peripheral blood samples from 120 participants were collected in PAXgene Blood RNA Tubes (Qiagen, Valencia, California) at each post-work exam. Detailed data processing procedures can be found in the Supplemental Materials. Briefly, total RNA was extracted using the PAXgene Blood-RNA Kit Qiagen-763134 (Qiagen, Valencia, California). All samples had optical density ratios of 280/260 ≥1.9 and 260/230 ≥1.8. RIN (RNA Integrity Number) scores, thus showing excellent RNA quality (mean: 8.3±0.9). We profiled miRNAs using NanoStringnCounter-miRNA expression analysis (NanoString Technologies, Seattle, Washington). The nCounter miRNA data were also confirmed through cross-platform validation in 20 randomly-selected study samples using the TaqManOpenArray Real-Time PCR Plates (Life Technologies, Carlsbad, California) on the QuantStudio 12K Flex Real-Time PCR System. The average Pearson correlation coefficient was 0.73 (0.63–0.79) between the two platforms, thus confirming the robustness of nCounter. The raw and processed miRNA data have been deposited into the NCBI Gene Expression Omnibus (accession number GSE63087). miRNA expression data were processed and obtained as described previously [24]. After discarding miRNAs that were not detectable in over 90% of samples, 166 miRNAs (including seven viral miRNAs) were retained for analysis.

### Blood pressure measurements

The seated BP of each individual was measured by a trained research assistant at each examination after a full five minutes of rest. Per the American Heart Association’s standardized measurement protocol[29], BP was measured via mercury sphygmomanometer on the right arm using an appropriate cuff size and three readings separated by at least one minute taken. BP was calculated from the average of the second and third readings and rounded up to the nearest whole number. Mean arterial pressure (MAP) was estimated by adding 1/3 of the difference between systolic blood pressure (SBP) and diastolic blood pressure (DBP) to the DBP value.

### Statistical analysis

For our descriptive analysis we used mixed-effects linear regression models to assess each of the three BP measures (pre- and post-work) across categories of independent variables (continuous variables were categorized according to the distribution of the data) while incorporating repeated measures data. We also conducted t-tests to compare pre- and post-work BP measures, and generated MA plots of describing differential miRNA expression by pre-work BP level. Next we used mixed-effects linear regression models to evaluate the associations of miRNAs with BP in the entire sample (pooled analysis) accounting for repeated measures. We also conducted sensitivity analyses to explore the effect of using the change from pre- to post-work in each BP measurements as an additional outcome, and separate analyses of each visit.

Next, for our stratified analyses we identified gender, BMI, smoking status, and alcohol consumption as EBP risk factors based on our descriptive analysis (variables associated with BP at p<0.05). We performed stratified analyses on these risk factors as well as occupation (based on our prior work finding differential pollutant exposure by occupation [30]) to explore potential differential miRNAs expression. For the BMI-stratified analysis we selected a cut-point of 23kg/m^2^ based on WHO recommendations for BMI measurement and intervention in Asian populations [31]. For the pooled analysis, the regression model was adjusted for covariates including age, occupation, gender, BMI, smoking status, number of cigarettes smoked on examination day, examination date, alcohol consumption, work hours per week, and outdoor temperature and dew point on the examination day. In the stratified analyses, we adjusted for all variables listed above except the stratification variable (occupation, sex, BMI, smoking status, and usual alcohol drinking). Because our previous findings suggested that air pollutant exposures impact BP [23], we also adjusted for personal particulate matter ≤10 μm (PM_10_). Since miRNA data was log-2 transformed we presented the results as unit change in mmHg of BP per each two-fold increase in miRNA. All statistical tests were two-sided, and BP changes with a Benjamini-Hochberg false discovery rate (FDR) <5% [32] were considered statistically significant. All analyses were performed using SAS 9.4 (Cary, NC).

## Results

### Characteristics of study participants and blood pressure measurements

Blood pressure measures before and after work by participant characteristics are summarized in Table 1 and Table 2, respectively. Briefly SBP, DBP, and MAP all significantly differed across gender, BMI, smoking status, and alcohol consumption at both times of day. In addition, all three post-work BP measures varied across temperature, and SBP by dew point, on exam days. No BP measures significantly varied across age, occupation, or calendar day of exam. SBP was significantly higher at the post-work exam compared to pre-work (p<0.01), but DBP and MAP were not (p = 0.69 and 0.45, respectively) (S1 Fig).

**Table 1:** Pre-work blood pressure by participant characteristics.

Variables	N(%)	Systolic Blood Pressure			Diastolic Blood Pressure			Mean Arterial Pressure		
		Mean^a^	SE^a^	p-value^b^	Mean^a^	SE^a^	p-value^b^	Mean^a^	SE^a^	p-value^b^
**Group, n (%)**										
Office workers	120 (50%)	111.5	1.5	0.14	77.7	1.1	0.26	89.0	1.2	0.19
Truck drivers	120 (50%)	114.6	1.5		79.5	1.1		91.2	1.2	
**Sex**										
Female	80 (33.33%)	104.9	1.6	**<0.01**	73.6	1.3	**<0.01**	84.1	1.3	**<0.01**
Male	160 (66.67%)	116.8	1.1		81.0	0.9		92.9	0.9	
**Age (Quartile)**										
Q1 [18–27 years]	60 (25%)	112.1	2.1	0.39	77.7	1.6	0.12	89.2	1.7	0.15
Q2 [28–32 years]	62 (25.83%)	111.6	2.0		76.5	1.6		88.2	1.7	
Q3 [33–37 years]	58 (24.17%)	112.6	2.1		78.7	1.6		90.0	1.7	
Q4 [38–46 years]	60 (25%)	116.2	2.1		81.8	1.6		93.4	1.7	
**BMI**										
≤23 kg/m^2^	120 (50%)	108.2	1.3	**<0.01**	74.7	1.0	**<0.01**	85.9	1.1	**<0.01**
>23 kg/m^2^	120 (50%)	118.6	1.3		82.6	1.0		94.6	1.1	
**Smoking habits**										
Never smoked	138 (57.5%)	109.7	1.3	**<0.01**	76.8	1.1	**0.01**	87.8	1.1	**<0.01**
Ever smoked	102 (42.5%)	117.4	1.5		80.9	1.2		93.1	1.3	
**Usual alcohol drinking**										
Yes	90 (37.5%)	110.0	1.2	**<0.01**	76.7	1.0	**<0.01**	87.9	1.0	**<0.01**
No	150 (62.5%)	117.8	1.6		81.6	1.3		93.7	1.3	
**Temperature** ^**c**^										
Low [20–25 °C]	110 (35.83%)	112.9	1.2	0.87	78.7	1.0	0.78	90.1	1.0	0.94
High [26–29 °C]	130 (54.17%)	113.1	1.1		78.5	0.9		90.1	0.9	
**Dew point** ^**c**^										
Low [16–20 °C]	107 (44.58%)	114.1	1.2	0.07	78.6	1.0	0.99	90.4	1.0	0.50
High [21–24 °C]	133 (55.42%)	112.2	1.1		78.6	0.9		89.9	0.9	
**Day of the week**										
Monday	35 (14.58%)	112.1	1.6	0.10	79.0	1.4	0.46	90.1	1.3	0.28
Tuesday	31 (12.92%)	111.6	1.8		76.6	1.5		88.2	1.5	
Wednesday	29 (12.08%)	114.9	1.9		78.7	1.6		90.7	1.6	
Thursday	35 (14.58%)	110.9	1.8		76.7	1.5		88.2	1.5	
Friday	36 (15%)	112.0	1.9		79.3	1.6		90.2	1.5	
Saturday	34 (14.17%)	113.1	2.0		79.0	1.7		90.6	1.7	
Sunday	40 (16.67%)	117.0	1.7		80.3	1.4		92.5	1.4	

^a^ Means and standard error of blood pressure and heart rate measured on two examination days were estimated by marginal means and corresponding standard error from mixed-effects regression models.

^b^ p-values were calculated using mixed-effects regression models.

^c^ Temperature and dew point were measured on the study examination day.

**Table 2:** Post-work blood pressure by participant characteristics.

Variables	N(%)	Systolic Blood Pressure			Diastolic Blood Pressure			Mean Arterial Pressure		
		Mean^a^	SE^a^	p-value^b^	Mean^a^	SE^a^	p-value^b^	Mean^a^	SE^a^	p-value^b^
**Group, n (%)**										
Office workers	120 (50%)	115.3	1.5	0.66	77.8	1.1	0.10	90.3	1.2	0.23
Truck drivers	120 (50%)	116.3	1.5		80.3	1.1		92.3	1.2	
**Sex**										
Female	80 (33.33%)	107.8	1.7	**<0.01**	74.2	1.2	**<0.01**	85.4	1.3	**<0.01**
Male	160 (66.67%)	119.9	1.2		81.6	0.9		94.2	0.9	
**Age (Quartile)**										
Q1 [18–27 years]	60 (25%)	116.9	2.2	0.83	78.0	1.5	0.40	90.9	1.7	0.71
Q2 [28–32 years]	62 (25.83%)	114.8	2.2		77.6	1.5		90.0	1.6	
Q3 [33–37 years]	58 (24.17%)	114.8	2.2		80.2	1.5		91.7	1.7	
Q4 [38–46 years]	60 (25%)	116.8	2.2		80.5	1.5		92.6	1.7	
**BMI**										
≤23 kg/m^2^	120 (50%)	111.6	1.4	**<0.01**	75.5	1.0	**<0.01**	87.5	1.1	**<0.01**
>23 kg/m^2^	120 (50%)	120.0	1.4		82.6	1.0		95.0	1.1	
**Smoking habits**										
Never smoked	138 (57.5%)	112.6	1.4	**<0.01**	77.6	1.0	**0.02**	89.2	1.1	**<0.01**
Ever smoked	102 (42.5%)	120.1	1.6		81.1	1.1		94.0	1.2	
**Usual alcohol drinking**										
Yes	90 (37.5%)	113.0	1.3	**<0.01**	77.1	0.9	**<0.01**	89.0	1.0	**<0.01**
No	150 (62.5%)	120.6	1.7		82.4	1.2		95.1	1.3	
**Temperature** ^**c**^										
Low [20–25 °C]	110 (35.83%)	117.3	1.2	**0.01**	80.3	0.9	**0.01**	92.5	0.9	**<0.01**
High [26–29 °C]	130 (54.17%)	114.6	1.2		78.1	0.8		90.2	0.9	
**Dew point** ^**c**^										
Low [16–20 °C]	107 (44.58%)	117.1	1.2	**0.02**	79.2	0.9	0.86	91.8	1.0	0.30
High [21–24 °C]	133 (55.42%)	114.8	1.2		79.0	0.8		90.9	0.9	
**Day of the week**										
Monday	35 (14.58%)	115.7	1.7	0.73	78.0	1.3	0.54	90.6	1.3	0.59
Tuesday	31 (12.92%)	115.6	1.8		77.4	1.4		90.0	1.4	
Wednesday	29 (12.08%)	116.9	1.9		79.1	1.5		91.5	1.5	
Thursday	35 (14.58%)	116.9	1.8		80.8	1.4		93.0	1.4	
Friday	36 (15%)	113.2	1.9		78.7	1.5		90.2	1.5	
Saturday	34 (14.17%)	116.1	2.0		79.0	1.6		91.4	1.6	
Sunday	40 (16.67%)	116.4	1.7		80.1	1.4		92.0	1.4	

^a^ Means and standard error of blood pressure and heart rate measured on two examination days were estimated by marginal means and corresponding standard error from mixed-effects regression models.

^b^ p-values were calculated using mixed-effects regression models.

^c^ Temperature and dew point were measured on the study examination day.

### Pooled analyses

**Fig 1:**
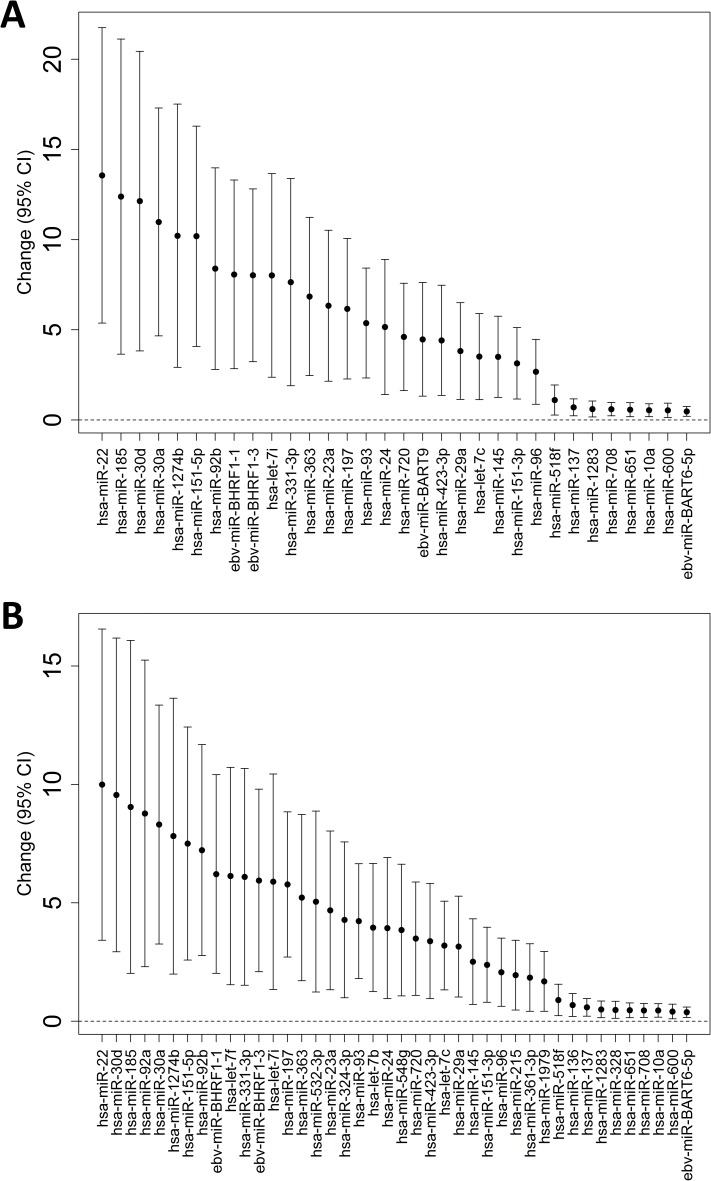
miRNAs associated with pre-work blood pressure changes in pooled analysis. Significant miRNAs associated with (A) SBP, and (B) MAP at FDR<5% in the pooled analysis.

Pre-work BP measured was significantly associated with post-work miRNA expression (Table 3). We identified 43 miRNAs whose levels were associated with one or more BP measures: 32 with SBP and 42 with MAP (Table 3, S1 and S2 Tables). No associations were identified for DBP. Fig 1A and 1B show miRNA associations with pre-work SBP and MAP, respectively, arranged by magnitude of unit difference in mmHg per each two-fold increase in miRNA. We did not find any significant associations of miRNA expression levels with post-work BP, nor with BP change from pre- to post-work measures. Analyzing each examination day separately attenuated the statistical significance of all findings (due to loss of sample size), but in general results were similar in direction and magnitude to those of our pooled, mixed-model analysis (data available upon request). Our MA plots of pre-work BP can also be found in our supplementary materials (S2 Fig).

**Table 3:** Number of miRNAs associated with BP measured pre- and post-work, in all participants and by strata of occupation and characteristics at FDR <0.05.

	Pre-Work			Post-Work		
	Any BP Measure	All BP Measures	Stratum-Specific miRNAs	Any BP Measure	All BP Measures	Stratum-Specific miRNAs
**All Participants**	43	0	N/A	0	0	N/A
**Occupation**						
Office workers	0	0	0	0	0	0
Truck drivers	39	0	7	0	0	0
**Sex**						
Female	0	0	0	0	0	0
Male	0	0	0	0	0	0
**BMI**						
≤23kg/m^2^ (Low)	0	0	0	0	0	0
>23kg/m^2^ (High)	60	0	17	0	0	0
**Smoking Status**						
Never smoked	0	0	0	0	0	0
Ever smoked	0	0	0	4	0	4
**Usual alcohol drinking**						
No	0	0	0	0	0	0
Yes	10	0	1	0	0	0

### Stratified analyses

For the stratified analyses we found significant associations between miRNAs and pre-work BP in truck drivers, high-BMI participants, and usual alcohol drinkers (Table 3, S1 and S2 Tables). In truck drivers 39 miRNAs were associated with SBP, of which seven were unique to the truck driver stratum. In high-BMI participants 60 miRNAs were associated with SBP including 18 miRNAs that were unique to participants in the high-BMI stratum. In usual alcohol drinkers 10 miRNAs were associated with MAP, including one miRNA unique to the usual alcohol drinkers stratum. For the post-work BP measures we identified four miRNAs associated with MAP in ever smokers (S3 Table), all of which were unique to the stratum of ever smokers.

## Discussion

To our knowledge this study is the first to evaluate miRNA profiling from blood leukocytes in relation to BP among a cohort of Chinese workers. We observed that both pre- and post-work SBP, DBP, and MAP were all higher in participants that were male, had higher BMI, or were smokers; all traditional risk factors of HTN [33]. Our pooled analysis also revealed 43 miRNAs associated with one or more BP measures but only at the pre-work exams. The stratified analyses showed varied significant associations between miRNAs and pre-work BP measures by strata of subject characteristics including occupation, BMI, and usual alcohol drinking, as well as only ever-smokers had a panel of miRNAs significantly associated with post-work BP.

Previous studies have related some miRNAs to molecular changes involved with HTN or cardiovascular diseases. For instance, we identified miR-151-5p as one of the most significant positive miRNA-BP associations in our pooled analysis as well as the strata of truck drivers, high-BMI participants, and usual alcohol drinkers (Supplemental Tables 1–2). A previous study linked down-regulation of miR-151-5p to ischemic arrhythmia [34], while a second linked increased miR-151-5p expression to aneurysm prevalence [35]. As both of these outcomes are potentially linked to HTN, miR-151-5p could be an early sign of HTN and thus a potentially useful early detection biomarker for HTN and/or a range of cardiovascular diseases. For another primary result of our pooled analysis (and the stratum of truck drivers), miR-22, systemic administration of miR-22 antagomirs reduced BP in spontaneously hypertensive rats [36], suggesting its potential therapeutic value for hypertensive patients. Our findings add to the evidence of a role for these miRNAs in the pathophysiology of HTN.

miRNA expression levels can be affected by a large number of factors and regulatory processes [37]. The distinct groups of miRNAs associated with EBP within strata of various subject characteristics therefore suggest potential molecular pathways for BP-related risk factors. For instance, miR-425 was found to be positively associated with BP in our high-BMI group (and neither our pooled analysis nor our other strata), and has been linked to blood pressure regulation through salt homeostasis (preventing its binding with appropriate gene) [38]. This finding may therefore be a biomarker of higher salt consumption in the high-BMI stratum. Another example in our high-BMI stratum, miR-208b, regulates the expression of slow myosin and thus plays an important role in both exercising and stress-response, and both of which are involved in the progression of EBP to HTN [39, 40]. These and other miRNAs identified in our high-BMI stratum should be examined in future research for associations with salt intake or other lifestyle factors (particularly diet) known to be associated with both BMI and HTN. These results could indicate miRNA involvement in the well-characterized relationship between high BMI and HTN.

Furthermore several miRNAs identified as significant in both our pooled and stratified analyses have been associated with pulmonary hypertension and/or traditional cardiovascular risk factors in prior epidemiological studies. miR-96 has been previously associated with pulmonary hypertension via 5-HT1BR expression; [41] while we lacked the data necessary to explore this pathway in our analysis, we also found miR-96 was significantly associated with BP measures. Bye et al. [42] also found that circulating miR-21 in men was negatively associated with aerobic fitness (itself associated with BP) while Wei et al. [22] found that miR-21 was associated with pulmonary hypertension along with two of our other significant results (miRs 191and 208b). While our results are based on miRNA expression in blood leukocytes, and are therefore not necessarily directly related to the circulating miRNAs reported in the literature, the overlap in these results (particularly given the racial differences between our cohort and those reported previously) are suggestive of common pathways in the interplay between the traditional risk factors of occupation, BMI, and smoking status and EBP development that should be explored in the future.

The fact that the majority of our findings are present only in the pre-work rather than the post-work BP measurements is curious, but perhaps unsurprising. BP is known to vary during the day [26, 27], and a morning surge in BP is a risk factor for cardiovascular disease and other adverse health outcomes [43]. A morning BP surge has also been associated with BP-related risk factors, including several identified as associated with specific pools of miRNAs in our study (e.g., alcohol use and smoking) [43]. Conversely, some attempts to replicate these links with a morning blood pressure surge have been unsuccessful with poor reproducibility and various subject-specific factors (e.g., sleep quality) cited as potentially confounding factors [44]. Given that cardiovascular events also tend to occur more frequently and BP in general tends to be higher after awakening [45], measurements of BP after this time (e.g., to account for between-subject sleep quality) may be a more reproducible health predictor. The fact that most of our stratified analyses were significant only at the pre-work measurement also raises the possibility that other unmeasured confounders occurring during the work day (e.g., diet, stress, physical activity) affect BP measurements, and that later studies of post-translational gene expression modification in HTN would be best served by measuring BP-related outcomes early in the day.

Our study is subject to a number of limitations. Our small sample size limited our ability to make statistical inferences, and cannot establish temporality. The high air pollution exposure in our population could attenuate associations that operate on competing molecular pathways. We attempted to address this limitation by controlling for air pollution exposure in our study, but nonetheless our results may have false negatives. In addition data on white blood cell counts and abundancies were not collected in this cohort, which limits our ability to draw biological or mechanistic insight from these findings. We attempted to minimize potential confounding due to short-term changes in blood cell abundancies[46] by employing a repeated-measures, matched study design with mixed effect models. As intra-individual miRNA and gene expression profiles are relatively stable over short (<1 year) time scales [47], any short-term inter- or intra-individual changes in blood composition are likely to be very small and unlikely to change our results substantively. Exploring miRNAs as potential blood-based biomarkers will still facilitate subsequent research in cohorts without contemporaneous blood composition data, and prior biomarker studies that did not include it [30, 48–53]. Finally, all of our significant associations were in the same direction, a potentially unusual finding. This could be due to residual confounding, or ‘morning surge’ in BP discussed above. However given the many miRNAs we simultaneously analyzed, there is also the possibility that this is a new finding. If miRNAs are largely up-regulated in response to increasing BP, global miRNA levels could be a useful biomarker. Further research in diverse populations using a similar, repeated measures design should be conducted to further explore these possibilities.

Despite these limitations, our study has notable strengths. The homogeneity of our population allows a limited control of certain unmeasured confounding factors: a small, racially-homogenous group living in the same area would be expected to have similar dietary and environmental exposures, serving as a crude form of matching that could reduce the effects of a variety of unmeasured confounders. In addition, our use of multiple measurements for both BP and miRNAs allowed us to account for short-term variations in both factors, making our results more likely to reflect trends that are of greater public health relevance.

Our results reveal miRNAs significantly associated with BP, many of them exclusive to strata known to be high-risk for HTN. Future research in diverse cohorts is needed to validate these findings, but if confirm these results may shed light on the development of a miRNA panel for early detection of HTN (and potentially other cardiovascular outcomes) in the future and highlighting the potential for miRNA-mediated risk factors for HTN and other cardiovascular diseases. Future research should follow these results using larger, more representative populations to identify specific pathways in humans linking the functions of these miRNAs to their role in elevating BP.

## Supporting information

S1 Table Significant changes in pre-work SBP (mmHg) per two-fold increase in miRNA expression level. (PDF)

S2 Table Significant changes in pre-work MAP (mmHg) per two-fold increase in miRNA expression level. (PDF)

S3 Table Significant changes in post-work MAP (mmHg) per two-fold increase in miRNA expression level. (PDF)

S1 Fig Box plots and t-test results comparing pre- and post-work BP measurements. (PDF)

S2 Fig MA plots of differential miRNA expression by BP level. (PDF)

## References

[1] A global brief on hypertension: silent killer, global public health crisis World Health Organization; 2013.

[2] Mozaffarian D, Benjamin EJ, Go AS, Arnett DK, Blaha MJ, Cushman M, et al Heart Disease and Stroke Statistics-2016 Update: A Report From the American Heart Association. Circulation. 2016;133(4):e38–e360. 10.1161/CIR.0000000000000350 26673558

[3] Nwankwo T, Yoon SS, Burt V, Gu Q. Hypertension among adults in the United States: National Health and Nutrition Examination Survey, 2011–2012. NCHS data brief. 2013(133):1–8. 24171916

[4] Wang W, Hu SS, Kong LZ, Gao RL, Zhu ML, Wang WY, et al Summary of report on cardiovascular diseases in China, 2012. Biomed Environ Sci. 2014;27(7):552–8. 10.3967/bes2014.085 25073915

[5] Jiang J, Liu M, Troy LM, Bangalore S, Hayes RB, Parekh N. Concordance with DASH diet and blood pressure change: results from the Framingham Offspring Study (1991–2008). Journal of hypertension. 2015;33(11):2223–30. 10.1097/HJH.0000000000000710 26259122

[6] Wong MC, Wang HH, Kwan MW, Fong BC, Chan WM, Zhang de X, et al Dietary counselling has no effect on cardiovascular risk factors among Chinese Grade 1 hypertensive patients: a randomized controlled trial. European heart journal. 2015;36(38):2598–607. 10.1093/eurheartj/ehv329 26264550

[7] Wang Z, Neuburg D, Li C, Su L, Kim JY, Chen JC, et al Global gene expression profiling in whole-blood samples from individuals exposed to metal fumes. Environ Health Perspect. 2005;113(2):233–41. 10.1289/txg.7273 15687063 PMC1277870

[8] Dubowsky SD, Suh H, Schwartz J, Coull BA, Gold DR. Diabetes, obesity, and hypertension may enhance associations between air pollution and markers of systemic inflammation. Environ Health Perspect. 2006;114(7):992–8. 10.1289/ehp.8469 16835049 PMC1513328

[9] Calderon-Garciduenas L, Villarreal-Calderon R, Valencia-Salazar G, Henriquez-Roldan C, Gutierrez-Castrellon P, Torres-Jardon R, et al Systemic inflammation, endothelial dysfunction, and activation in clinically healthy children exposed to air pollutants. Inhalation toxicology. 2008;20(5):499–506. 10.1080/08958370701864797 18368620

[10] Cesari M, Penninx BW, Newman AB, Kritchevsky SB, Nicklas BJ, Sutton-Tyrrell K, et al Inflammatory markers and cardiovascular disease (the health, aging and body composition [health ABC] study). The American journal of cardiology. 2003;92(5):522–8. 12943870 10.1016/s0002-9149(03)00718-5

[11] Role of epigenetics in blood pressure regulation and development of hypertension. National Heart, Lung, and Blood Institue: National Institute of Health (NIH). 2012.

[12] Millis RM. Epigenetics and hypertension. Current hypertension reports. 2011;13(1):21–8. 10.1007/s11906-010-0173-8 21125351

[13] Raftopoulos L, Katsi V, Makris T, Tousoulis D, Stefanadis C, Kallikazaros I. Epigenetics, the missing link in hypertension. Life sciences. 2015;129:22–6. 10.1016/j.lfs.2014.08.003 25128856

[14] Weinhold B. Epigenetics: the science of change. Environmental Health Perspectives. 2006;114(3):A160 16507447 10.1289/ehp.114-a160 PMC1392256

[15] Fabian MR, Sonenberg N, Filipowicz W. Regulation of mRNA translation and stability by microRNAs. Annu Rev Biochem. 2010;79:351–79. 10.1146/annurev-biochem-060308-103103 20533884

[16] Winter J, Jung S, Keller S, Gregory RI, Diederichs S. Many roads to maturity: microRNA biogenesis pathways and their regulation. Nature cell biology. 2009;11(3):228–34. 10.1038/ncb0309-228 19255566

[17] Baccarelli A, Bollati V. Epigenetics and environmental chemicals. Curr Opin Pediatr. 2009;21(2):243–51. 19663042 10.1097/mop.0b013e32832925cc PMC3035853

[18] He L, Hannon GJ. MicroRNAs: small RNAs with a big role in gene regulation. Nat Rev Genet. 2004;5(7):522–31. 10.1038/nrg1379 15211354

[19] Zhou G, Chen T, Raj JU. MicroRNAs in pulmonary arterial hypertension. Am J Respir Cell Mol Biol. 2015;52(2):139–51. 10.1165/rcmb.2014-0166TR 25192340 PMC4370247

[20] Bertero T, Cottrill K, Krauszman A, Lu Y, Annis S, Hale A, et al The microRNA-130/301 family controls vasoconstriction in pulmonary hypertension. J Biol Chem. 2015;290(4):2069–85. 10.1074/jbc.M114.617845 25505270 PMC4303661

[21] Xu J, Zhao J, Evan G, Xiao C, Cheng Y, Xiao J. Circulating microRNAs: novel biomarkers for cardiovascular diseases. J Mol Med (Berl). 2012;90(8):865–75. 22159451 10.1007/s00109-011-0840-5

[22] Wei C, Henderson H, Spradley C, Li L, Kim IK, Kumar S, et al Circulating miRNAs as potential marker for pulmonary hypertension. PLoS One. 2013;8(5):e64396 10.1371/journal.pone.0064396 23717609 PMC3662705

[23] Baccarelli A, Barretta F, Dou C, Zhang X, McCracken JP, Diaz A, et al Effects of particulate air pollution on blood pressure in a highly exposed population in Beijing, China: a repeated-measure study. Environ Health-Glob. 2011;10. 10.1186/1476-069X-10-108 PMC3273442 22188661

[24] Hou L, Barupal J, Zhang W, Zheng Y, Liu L, Zhang X, et al Particulate Air Pollution Exposure and Expression of Viral and Human MicroRNAs in Blood: The Beijing Truck Driver Air Pollution Study. Environ Health Perspect. 2015. 10.1289/ehp.1408519 PMC4786978 26068961

[25] Baccarelli A, Barretta F, Dou C, Zhang X, McCracken JP, Diaz A, et al Effects of particulate air pollution on blood pressure in a highly exposed population in Beijing, China: a repeated-measure study. Environ Health. 2011;10:108 10.1186/1476-069X-10-108 22188661 PMC3273442

[26] Millar-Craig MW, Bishop CN, Raftery EB. Circadian variation of blood-pressure. Lancet (London, England). 1978;1(8068):795–7. 10.1016/s0140-6736(78)92998-7 85815

[27] Mancia G, Ferrari A, Gregorini L, Parati G, Pomidossi G, Bertinieri G, et al Blood pressure and heart rate variabilities in normotensive and hypertensive human beings. Circ Res. 1983;53(1):96–104. 6861300 10.1161/01.res.53.1.96

[28] National Climatic Data Center (U.S.). NNDC climate data online. Production ed. [Asheville, N.C.]: National Climatic Data Center].

[29] Pickering TG, Hall JE, Appel LJ, Falkner BE, Graves J, Hill MN, et al Recommendations for blood pressure measurement in humans and experimental animals: Part 1: blood pressure measurement in humans: a statement for professionals from the Subcommittee of Professional and Public Education of the American Heart Association Council on High Blood Pressure Research. Hypertension. 2005;45(1):142–61. 10.1161/01.HYP.0000150859.47929.8e 15611362

[30] Hou L, Barupal J, Zhang W, Zheng Y, Liu L, Zhang X, et al Particulate Air Pollution Exposure and Expression of Viral and Human MicroRNAs in Blood: The Beijing Truck Driver Air Pollution Study. Environ Health Perspect. 2016;124(3):344–50. 10.1289/ehp.1408519 26068961 PMC4786978

[31] Appropriate body-mass index for Asian populations and its implications for policy and intervention strategies. Lancet (London, England). 2004;363(9403):157–63. 10.1016/S0140-6736(03)15268-3 14726171

[32] Benjamini Y, Hochberg Y. Controlling the False Discovery Rate: A Practical and Powerful Approach to Multiple Testing. Journal of the Royal Statistical Society Series B (Methodological). 1995;57(1):289–300.

[33] Risk Factors for High Blood Pressure. National Heart, Lung, and Blood Institue: National Institute of Health (NIH). 2015.

[34] Zhang Y, Wang R, Du W, Wang S, Yang L, Pan Z, et al Downregulation of miR-151-5p contributes to increased susceptibility to arrhythmogenesis during myocardial infarction with estrogen deprivation. PLoS One. 2013;8(9):e72985 10.1371/journal.pone.0072985 24039836 PMC3767733

[35] Li PX, Zhang QY, Wu X, Yang XJ, Zhang Y, Li YX, et al Circulating microRNAs Serve as Novel Biological Markers for Intracranial Aneurysms. J Am Heart Assoc. 2014;3(5). 10.1161/JAHA.114.000972 PMC4323791 25249297

[36] Friese RS, Altshuler AE, Zhang K, Miramontes-Gonzalez JP, Hightower CM, Jirout ML, et al MicroRNA-22 and promoter motif polymorphisms at the Chga locus in genetic hypertension: functional and therapeutic implications for gene expression and the pathogenesis of hypertension. Hum Mol Genet. 2013;22(18):3624–40. 10.1093/hmg/ddt213 23674521 PMC3749858

[37] Cui K, Lyu Q, Xu N, Liu Q, Zhang J, Xing W, et al Characterization of the microRNA pool and the factors affecting its regulatory potential. Integr Biol (Camb). 2014;6(12):1141–52. 25222482 10.1039/c4ib00156g

[38] Arora P, Wu C, Khan AM, Bloch DB, Davis-Dusenbery BN, Ghorbani A, et al Atrial natriuretic peptide is negatively regulated by microRNA-425. J Clin Invest. 2013;123(8):3378–82. 10.1172/JCI67383 23867623 PMC3726159

[39] Neves VJ, Fernandes T, Roque FR, Soci UP, Melo SF, de Oliveira EM. Exercise training in hypertension: Role of microRNAs. World J Cardiol. 2014;6(8):713–27. 10.4330/wjc.v6.i8.713 25228951 PMC4163701

[40] van Rooij E, Quiat D, Johnson BA, Sutherland LB, Qi XX, Richardson JA, et al A Family of microRNAs Encoded by Myosin Genes Governs Myosin Expression and Muscle Performance. Dev Cell. 2009;17(5):662–73. 10.1016/j.devcel.2009.10.013 19922871 PMC2796371

[41] Wallace E, Morrell NW, Yang XD, Long L, Stevens H, Nilsen M, et al A Sex-Specific MicroRNA-96/5-Hydroxytryptamine 1B Axis Influences Development of Pulmonary Hypertension. American journal of respiratory and critical care medicine. 2015;191(12):1432–42. 10.1164/rccm.201412-2148OC 25871906 PMC4476563

[42] Bye A, Rosjo H, Aspenes ST, Condorelli G, Omland T, Wisloff U. Circulating microRNAs and aerobic fitness—the HUNT-Study. PLoS One. 2013;8(2):e57496 10.1371/journal.pone.0057496 23469005 PMC3585333

[43] Kario K. Morning Surge in Blood Pressure and Cardiovascular Risk Evidence and Perspectives. Hypertension. 2010;56(5):765–73. 10.1161/HYPERTENSIONAHA.110.157149 20937968

[44] Asayama K, Wei FF, Hara A, Hansen TW, Li Y, Staessen JA. Prognosis in relation to blood pressure variability: con side of the argument. Hypertension. 2015;65(6):1170–9; discussion 9. 10.1161/HYPERTENSIONAHA.115.04808 25916728

[45] Kario K. Prognosis in relation to blood pressure variability: pro side of the argument. Hypertension. 2015;65(6):1163–9; discussion 9. 10.1161/HYPERTENSIONAHA.115.04800 25916727

[46] Holgate ST, Sandstrom T, Frew AJ, Stenfors N, Nordenhall C, Salvi S, et al Health effects of acute exposure to air pollution. Part I: Healthy and asthmatic subjects exposed to diesel exhaust. Research report (Health Effects Institute). 2003(112):1–30; discussion 51–67. 14738208

[47] Tabassum R, Sivadas A, Agrawal V, Tian H, Arafat D, Gibson G. Omic personality: implications of stable transcript and methylation profiles for personalized medicine. Genome medicine. 2015;7:88 10.1186/s13073-015-0209-4 26391122 PMC4578259

[48] Hou L, Zhang X, Dioni L, Barretta F, Dou C, Zheng Y, et al Inhalable particulate matter and mitochondrial DNA copy number in highly exposed individuals in Beijing, China: a repeated-measure study. Particle and fibre toxicology. 2013;10:17 10.1186/1743-8977-10-17 23628000 PMC3649952

[49] Hou L, Zhang X, Zheng Y, Wang S, Dou C, Guo L, et al Altered methylation in tandem repeat element and elemental component levels in inhalable air particles. Environmental and molecular mutagenesis. 2014;55(3):256–65. 10.1002/em.21829 24273195 PMC4001244

[50] Liu C, Xu J, Chen Y, Guo X, Zheng Y, Wang Q, et al Characterization of genome-wide H3K27ac profiles reveals a distinct PM2.5-associated histone modification signature. Environ Health. 2015;14:65 10.1186/s12940-015-0052-5 26276146 PMC4537530

[51] Ma L, Bai Y, Pu H, Gou F, Dai M, Wang H, et al Histone Methylation in Nickel-Smelting Industrial Workers. PLoS One. 2015;10(10):e0140339 10.1371/journal.pone.0140339 26474320 PMC4608576

[52] Sanchez-Guerra M, Zheng Y, Osorio-Yanez C, Zhong J, Chervona Y, Wang S, et al Effects of particulate matter exposure on blood 5-hydroxymethylation: results from the Beijing truck driver air pollution study. Epigenetics. 2015;10(7):633–42. 10.1080/15592294.2015.1050174 25970091 PMC4623004

[53] Zheng Y, Sanchez-Guerra M, Zhang Z, Joyce BT, Zhong J, Kresovich JK, et al Traffic-derived particulate matter exposure and histone H3 modification: A repeated measures study. Environmental research. 2017;153:112–9. 10.1016/j.envres.2016.11.015 27918982 PMC5605137

