# Supplementary material for: Blood pressure and expression of microRNAs in whole blood
Source: PLoS One. 2017 Mar 9;12(3):e0173550. doi: 10.1371/journal.pone.0173550 (PMC5344460; doi:10.1371/journal.pone.0173550)
Supplement: S1 Table — (PDF) [file pone.0173550.s001.pdf]

S1 Table Significant changes in pre-work SBP (mmHg) per one-fold increase in miRNA expression level.

miRNA	Change	95% CI		*P-value*	FDR*
Pooled analysis					
hsa-miR-93	5.37	2.33	8.42	<0.01	0.03
hsa-miR-30a	10.98	4.66	17.30	<0.01	0.03
ebv-miR-BART6-5p	0.47	0.20	0.75	<0.01	0.03
ebv-miR-BHRF1-3	8.02	3.23	12.81	<0.01	0.03
hsa-miR-151-5p	10.19	4.08	16.29	<0.01	0.03
hsa-miR-22	13.56	5.37	21.75	<0.01	0.03
hsa-miR-708	0.60	0.23	0.97	<0.01	0.03
hsa-miR-151-3p	3.14	1.16	5.12	<0.01	0.03
hsa-miR-197	6.16	2.27	10.06	<0.01	0.03
hsa-miR-363	6.85	2.46	11.23	<0.01	0.03
hsa-miR-145	3.50	1.25	5.75	<0.01	0.03
hsa-miR-720	4.60	1.63	7.58	<0.01	0.03
ebv-miR-BHRF1-1	8.07	2.84	13.30	<0.01	0.03
hsa-miR-23a	6.33	2.15	10.52	<0.01	0.03
hsa-miR-10a	0.54	0.18	0.90	<0.01	0.03
hsa-miR-92b	8.39	2.80	13.98	<0.01	0.03
hsa-miR-96	2.67	0.87	4.47	<0.01	0.03
hsa-miR-137	0.70	0.23	1.17	<0.01	0.03
hsa-let-7c	3.52	1.13	5.90	<0.01	0.03
hsa-miR-651	0.57	0.18	0.96	<0.01	0.03
hsa-miR-30d	12.14	3.83	20.44	<0.01	0.03
hsa-miR-423-3p	4.41	1.36	7.47	<0.01	0.04
hsa-miR-29a	3.82	1.14	6.50	0.01	0.04
hsa-let-7i	8.02	2.37	13.66	0.01	0.04
ebv-miR-BART9	4.47	1.32	7.62	0.01	0.04
hsa-miR-185	12.38	3.65	21.12	0.01	0.04
hsa-miR-1274b	10.21	2.92	17.51	0.01	0.04
hsa-miR-24	5.16	1.41	8.90	0.01	0.04
hsa-miR-1283	0.61	0.17	1.05	0.01	0.04
hsa-miR-600	0.54	0.14	0.93	0.01	0.04
hsa-miR-518f	1.11	0.27	1.94	0.01	0.05
hsa-miR-331-3p	7.64	1.90	13.39	0.01	0.05
**miRNA**	**Change**	**95% CI**		***P-value***	**FDR***
Truck drivers					
hsa-miR-22	16.34	5.24	27.44	<0.01	0.04
hsa-miR-1274b	12.86	3.45	22.27	0.01	0.04
hsa-miR-30a	12.60	3.94	21.26	0.01	0.04
hsa-miR-151-5p	12.08	3.89	20.27	<0.01	0.04

ebv-miR-BHRF1-3	11.62	4.06	19.17	<0.01	0.04
hsa-let-7i	11.09	3.31	18.88	0.01	0.04
hsa-miR-181a	10.67	2.69	18.65	0.01	0.04
hsa-miR-331-3p	10.51	3.21	17.81	0.01	0.04
ebv-miR-BHRF1-1	10.31	2.64	17.99	0.01	0.04
hsa-miR-23a	8.99	3.46	14.52	<0.01	0.04
hsa-miR-197	8.43	3.54	13.32	<0.01	0.04
hsa-miR-140-3p	8.31	1.91	14.71	0.01	0.05
hsa-miR-324-3p	7.57	2.39	12.76	<0.01	0.04
hiv1-miR-H1	7.33	1.69	12.97	0.01	0.05
hsa-miR-720	7.23	3.71	10.74	<0.01	0.01
hsa-miR-24	7.03	2.13	11.92	0.01	0.04
hsa-miR-548g	6.47	1.71	11.23	0.01	0.04
ebv-miR-BART9	6.46	1.98	10.95	0.01	0.04
hsa-miR-320a	5.92	1.75	10.09	0.01	0.04
hsa-miR-29a	5.41	1.58	9.23	0.01	0.04
hsa-miR-93	5.10	1.20	9.00	0.01	0.05
hsa-miR-145	4.69	2.31	7.06	<0.01	0.01
hsa-miR-183	4.34	1.19	7.49	0.01	0.04
hsa-miR-191	4.12	1.08	7.15	0.01	0.04
hsa-miR-1979	3.94	1.13	6.75	0.01	0.04
hsa-let-7c	3.84	0.98	6.70	0.01	0.04
hsa-miR-215	3.76	1.58	5.95	<0.01	0.04
hsa-miR-151-3p	3.69	1.28	6.11	<0.01	0.04
hsa-miR-361-3p	3.07	0.89	5.25	0.01	0.04
hsa-miR-96	3.02	0.71	5.34	0.01	0.05
hsa-miR-518f	1.38	0.39	2.37	0.01	0.04
hsa-miR-136	0.99	0.23	1.75	0.01	0.05
hsa-miR-137	0.84	0.23	1.45	0.01	0.04
hsa-miR-559	0.78	0.23	1.33	0.01	0.04
hsa-miR-1283	0.77	0.21	1.34	0.01	0.04
hsa-miR-651	0.72	0.20	1.25	0.01	0.04
hsa-miR-600	0.70	0.18	1.23	0.01	0.04
hsa-miR-708	0.65	0.18	1.13	0.01	0.04
ebv-miR-BART6-5p	0.54	0.18	0.91	<0.01	0.04

**miRNA**	**Change**	**95% CI**		***P-value***	**FDR***
High BMI					
hsa-miR-92a	27.44	14.02	40.85	<0.01	<0.01
hsa-miR-22	26.10	14.45	37.74	<0.01	<0.01
hsa-miR-30d	24.63	13.10	36.16	<0.01	<0.01
hsa-miR-151-5p	21.36	12.08	30.65	<0.01	<0.01
hsa-miR-186	20.20	8.99	31.42	<0.01	<0.01
hsa-miR-1274b	20.19	9.29	31.09	<0.01	<0.01

hsa-miR-30a	20.19	11.47	28.90	<0.01	<0.01
hsa-miR-30c	19.05	9.86	28.23	<0.01	<0.01
hsa-miR-425	18.63	7.34	29.91	<0.01	0.01
hsa-miR-185	18.16	5.37	30.94	0.01	0.02
hsa-miR-92b	15.92	6.45	25.39	<0.01	0.01
hsa-miR-331-3p	15.90	7.88	23.92	<0.01	<0.01
hsa-let-7i	15.41	7.19	23.62	<0.01	<0.01
hsa-miR-1975	14.43	4.34	24.51	0.01	0.02
hsa-miR-532-3p	13.86	6.73	20.98	<0.01	<0.01
ebv-miR-BHRF1-3	13.76	5.86	21.66	<0.01	0.01
ebv-miR-BHRF1-1	13.11	4.88	21.33	<0.01	0.01
hsa-miR-197	12.69	7.37	18.00	<0.01	<0.01
hsa-let-7f	12.44	3.78	21.11	0.01	0.02
hsa-miR-140-3p	12.34	4.91	19.77	<0.01	0.01
hsa-miR-148b	12.22	4.41	20.02	<0.01	0.01
hsa-miR-363	11.81	5.65	17.96	<0.01	<0.01
hsa-miR-23a	11.65	5.86	17.44	<0.01	<0.01
hsa-miR-24	10.15	4.69	15.61	<0.01	<0.01
hsa-miR-324-3p	9.52	3.45	15.59	<0.01	0.01
hsa-miR-423-3p	9.21	4.99	13.43	<0.01	<0.01
hsa-miR-93	9.12	4.98	13.25	<0.01	<0.01
hsa-miR-720	8.23	4.08	12.37	<0.01	<0.01
hsa-miR-29a	7.71	3.23	12.19	<0.01	0.01
hsa-let-7b	7.50	2.45	12.54	<0.01	0.01
hsa-miR-548g	7.33	2.36	12.30	<0.01	0.01
hsa-miR-320a	7.01	2.09	11.93	0.01	0.02
hsa-miR-145	6.64	2.33	10.94	<0.01	0.01
ebv-miR-BART9	6.48	1.98	10.99	0.01	0.02
hsa-let-7c	5.78	2.44	9.12	<0.01	0.01
hsa-miR-191	5.68	2.33	9.03	<0.01	0.01
hsa-miR-484	5.46	2.58	8.34	<0.01	<0.01
hsa-miR-1979	4.98	2.07	7.89	<0.01	0.01
hsa-miR-151-3p	4.83	1.92	7.73	<0.01	0.01
hsa-miR-361-3p	4.56	2.04	7.08	<0.01	<0.01
hsa-miR-96	4.36	1.71	7.02	<0.01	0.01
hsa-miR-584	3.99	0.96	7.03	0.01	0.03
hsa-miR-215	3.87	0.72	7.01	0.02	0.05
hsa-miR-1205	3.55	1.22	5.87	<0.01	0.01
hsa-miR-518f	2.07	0.84	3.31	<0.01	0.01
hsa-miR-1308	1.95	0.96	2.94	<0.01	<0.01
hsa-miR-208b	1.68	0.45	2.91	0.01	0.02
hsa-miR-195	1.44	0.30	2.57	0.01	0.04
hsa-miR-1183	1.34	0.43	2.25	<0.01	0.01

hsa-miR-136	1.32	0.41	2.23	0.01	0.02
hsa-miR-651	1.04	0.45	1.64	<0.01	0.01
hsa-miR-137	1.02	0.29	1.75	0.01	0.02
hsa-miR-1283	1.01	0.34	1.69	<0.01	0.01
hsa-miR-708	1.01	0.44	1.57	<0.01	0.01
hsa-miR-10a	0.97	0.44	1.51	<0.01	<0.01
hsa-miR-600	0.95	0.33	1.58	<0.01	0.01
hsa-miR-501-3p	0.94	0.26	1.61	0.01	0.02
hsa-miR-130b	0.94	0.38	1.49	<0.01	0.01
ebv-miR-BART6-5p	0.92	0.55	1.30	<0.01	<0.01
hsa-miR-328	0.91	0.30	1.52	<0.01	0.01
