# Supplementary material for: Blood pressure and expression of microRNAs in whole blood
Source: PLoS One. 2017 Mar 9;12(3):e0173550. doi: 10.1371/journal.pone.0173550 (PMC5344460; doi:10.1371/journal.pone.0173550)
Supplement: S2 Table — (PDF) [file pone.0173550.s002.pdf]

S2 Table Significant changes in pre-work MAP (mmHg) per one-fold increase in miRNA expression level.

miRNA	Change	95% CI		*P-value*	FDR*
Pooled analysis					
hsa-miR-22	9.99	3.42	16.56	<0.01	0.04
hsa-miR-30d	9.55	2.93	16.18	0.01	0.04
hsa-miR-185	9.05	2.02	16.08	0.01	0.05
hsa-miR-92a	8.77	2.30	15.25	0.01	0.04
hsa-miR-30a	8.31	3.26	13.35	<0.01	0.04
hsa-miR-1274b	7.82	1.99	13.64	0.01	0.04
hsa-miR-151-5p	7.50	2.58	12.42	<0.01	0.04
hsa-miR-92b	7.22	2.77	11.68	<0.01	0.04
ebv-miR-BHRF1-1	6.21	2.02	10.41	<0.01	0.04
hsa-let-7f	6.13	1.54	10.72	0.01	0.04
hsa-miR-331-3p	6.10	1.52	10.67	0.01	0.04
ebv-miR-BHRF1-3	5.94	2.09	9.80	<0.01	0.04
hsa-let-7i	5.89	1.34	10.44	0.01	0.05
hsa-miR-197	5.78	2.71	8.84	<0.01	0.04
hsa-miR-363	5.22	1.71	8.73	<0.01	0.04
hsa-miR-532-3p	5.05	1.23	8.87	0.01	0.04
hsa-miR-23a	4.68	1.33	8.03	0.01	0.04
hsa-miR-324-3p	4.28	0.99	7.57	0.01	0.05
hsa-miR-93	4.23	1.80	6.65	<0.01	0.04
hsa-let-7b	3.95	1.25	6.66	<0.01	0.04
hsa-miR-24	3.93	0.95	6.91	0.01	0.04
hsa-miR-548g	3.85	1.07	6.63	0.01	0.04
hsa-miR-720	3.49	1.09	5.88	<0.01	0.04
hsa-miR-423-3p	3.38	0.95	5.82	0.01	0.04
hsa-let-7c	3.19	1.32	5.07	<0.01	0.04
hsa-miR-29a	3.15	1.02	5.28	<0.01	0.04
hsa-miR-145	2.51	0.71	4.32	0.01	0.04
hsa-miR-151-3p	2.38	0.80	3.97	<0.01	0.04
hsa-miR-96	2.07	0.63	3.51	0.01	0.04
hsa-miR-215	1.94	0.47	3.42	0.01	0.04
hsa-miR-361-3p	1.84	0.42	3.27	0.01	0.05
hsa-miR-1979	1.68	0.42	2.94	0.01	0.04
hsa-miR-518f	0.90	0.23	1.56	0.01	0.04
hsa-miR-136	0.68	0.20	1.17	0.01	0.04
hsa-miR-137	0.59	0.21	0.96	<0.01	0.04
hsa-miR-1283	0.50	0.15	0.85	0.01	0.04
hsa-miR-328	0.48	0.12	0.84	0.01	0.04
hsa-miR-651	0.46	0.15	0.77	<0.01	0.04

hsa-miR-708	0.45	0.16	0.75	<0.01	0.04
hsa-miR-10a	0.45	0.17	0.74	<0.01	0.04
hsa-miR-600	0.41	0.10	0.72	0.01	0.05
ebv-miR-BART6-5p	0.38	0.16	0.60	<0.01	0.04

**miRNA**	**Change**	**95% CI**		***P-value***	**FDR***
Usual alcohol drinking					
hsa-miR-151-5p	12.97	5.52	20.42	<0.01	0.05
hsa-miR-92b	10.74	3.89	17.59	<0.01	0.05
hsa-miR-331-3p	10.71	4.31	17.11	<0.01	0.05
hsa-miR-363	9.01	3.34	14.68	<0.01	0.05
ebv-miR-BHRF1-1	8.87	3.41	14.33	<0.01	0.05
hsa-miR-151-3p	3.35	1.21	5.49	<0.01	0.05
hsa-miR-145	3.31	1.27	5.34	<0.01	0.05
hsa-miR-215	3.17	1.15	5.20	<0.01	0.05
hsa-miR-195	1.31	0.55	2.07	<0.01	0.05
hsa-miR-1283	0.94	0.37	1.52	<0.01	0.05
