# Supplementary material for: Blood pressure and expression of microRNAs in whole blood
Source: PLoS One. 2017 Mar 9;12(3):e0173550. doi: 10.1371/journal.pone.0173550 (PMC5344460; doi:10.1371/journal.pone.0173550)
Supplement: S3 Table — (PDF) [file pone.0173550.s003.pdf]

S3 Table Significant changes in post-work MAP (mmHg) per one-fold increase in miRNA expression level.

miRNA	Change	95% CI		*P-value*	FDR*
Ever smoked					
hsa-miR-92b	11.66	5.73	17.58	<0.01	0.04
ebv-miR-BHRF1-3	10.49	4.54	16.44	<0.01	0.04
hsa-miR-720	4.94	2.13	7.75	<0.01	0.04
hsa-miR-423-3p	4.68	1.97	7.40	<0.01	0.04
hsa-miR-1183	0.93	0.41	1.45	<0.01	0.04
