# Supplementary figures and images for: Blood pressure and expression of microRNAs in whole blood
Source: PLoS One. 2017 Mar 9;12(3):e0173550. doi: 10.1371/journal.pone.0173550 (PMC5344460; doi:10.1371/journal.pone.0173550)

**SBP**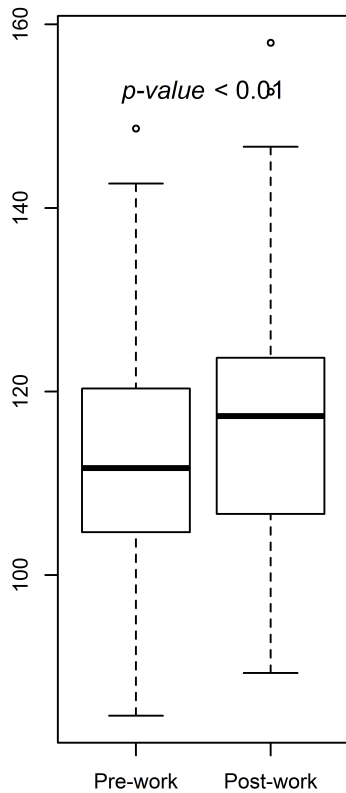**DBP**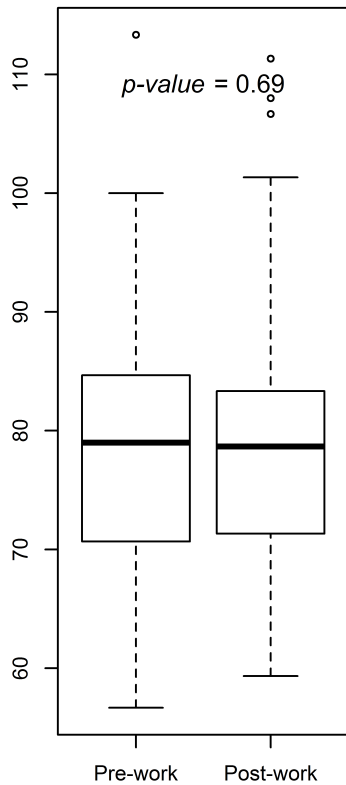**MAP**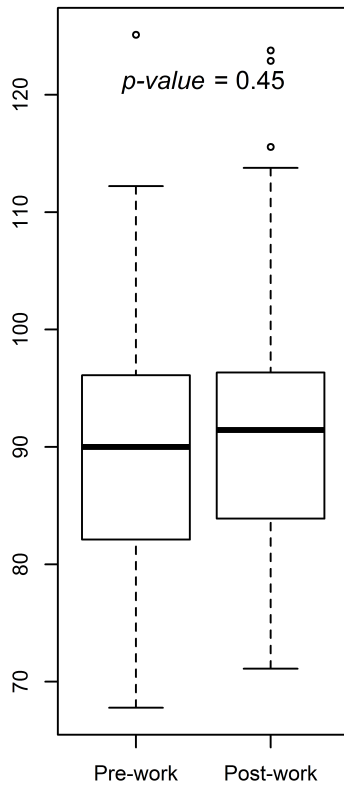

Supplement: S1 Fig — (PDF) [file pone.0173550.s004.pdf]
