# Supplementary material for: Blood pressure and expression of microRNAs in whole blood
Source: PLoS One. 2017 Mar 9;12(3):e0173550. doi: 10.1371/journal.pone.0173550 (PMC5344460; doi:10.1371/journal.pone.0173550)
Supplement: S2 Fig — (PDF) [file pone.0173550.s005.pdf]

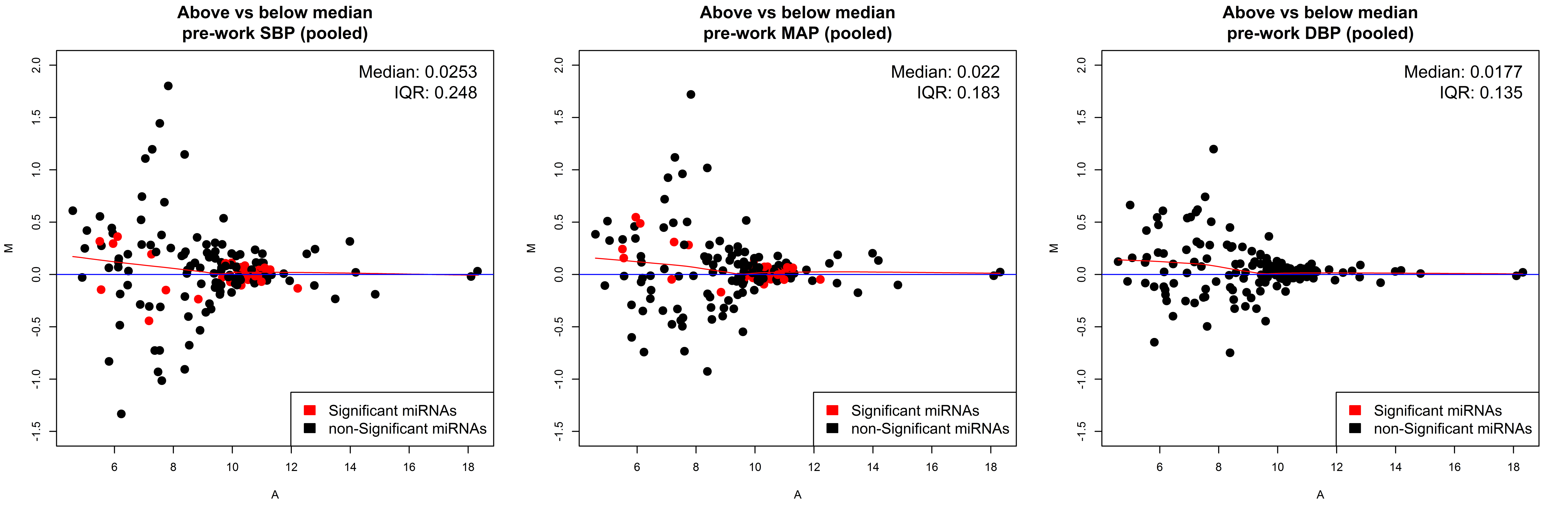
Above vs below median
pre-work SBP (pooled)
Median: 0.0253
IQR: 0.248
Significant miRNAs
non-Significant miRNAs
Above vs below median
pre-work MAP (pooled)
Median: 0.022
IQR: 0.183
Significant miRNAs
non-Significant miRNAs
Above vs below median
pre-work DBP (pooled)
Median: 0.0177
IQR: 0.135
Significant miRNAs
non-Significant miRNAs
M
A
